# Synthesis of DNA Origami Scaffolds: Current and Emerging Strategies

**DOI:** 10.3390/molecules25153386

**Published:** 2020-07-26

**Authors:** Joshua Bush, Shrishti Singh, Merlyn Vargas, Esra Oktay, Chih-Hsiang Hu, Remi Veneziano

**Affiliations:** 1 Volgenau School of Engineering, Department of Bioengineering, George Mason University, Fairfax, VA 22030, USA; jbush20@gmu.edu (J.B.); ssingh64@gmu.edu (S.S.); lvargasr@gmu.edu (M.V.); eoktay@gmu.edu (E.O.); chu6@gmu.edu (C.-H.H.); 2 Institute for Advanced Biomedical Research, George Mason University, Manassas, VA 20110, USA

**Keywords:** single-stranded DNA, DNA scaffolds, DNA origami, nucleic acid nanoparticles, DNA nanotechnology, DNA Synthesis, DNA amplification

## Abstract

DNA origami nanocarriers have emerged as a promising tool for many biomedical applications, such as biosensing, targeted drug delivery, and cancer immunotherapy. These highly programmable nanoarchitectures are assembled into any shape or size with nanoscale precision by folding a single-stranded DNA scaffold with short complementary oligonucleotides. The standard scaffold strand used to fold DNA origami nanocarriers is usually the M13mp18 bacteriophage’s circular single-stranded DNA genome with limited design flexibility in terms of the sequence and size of the final objects. However, with the recent progress in automated DNA origami design—allowing for increasing structural complexity—and the growing number of applications, the need for scalable methods to produce custom scaffolds has become crucial to overcome the limitations of traditional methods for scaffold production. Improved scaffold synthesis strategies will help to broaden the use of DNA origami for more biomedical applications. To this end, several techniques have been developed in recent years for the scalable synthesis of single stranded DNA scaffolds with custom lengths and sequences. This review focuses on these methods and the progress that has been made to address the challenges confronting custom scaffold production for large-scale DNA origami assembly.

## 1. Introduction

**Figure 1:**
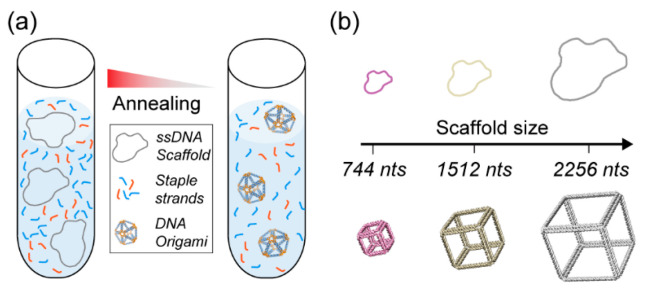
DNA origami nanoparticle assembly. (**a**) DNA origami are folded with a long single-stranded DNA scaffold and multiple staple strands via a thermal annealing process. (**b**) Controlling the DNA origami size with custom scaffold lengths.

In all known living organisms, DNA molecules are responsible for storing and carrying genetic information [1]. From a materials and biomedical engineering point of view, DNA molecules also represent a promising alternative to several natural and synthetic polymers that are typically used for biomedical applications such as drug delivery and cancer immunotherapy [2,3,4,5,6]. DNA-based materials differ from other polymeric materials as they offer programmability at the nanoscale, along with unique structural and biochemical properties [7,8]. These characteristics make them ideal as building blocks to assemble complex nanoarchitectures and further organize various biomolecules and inorganic molecules with nanometer-scale precision [7,8,9,10,11]. In vivo, DNA molecules are primarily present as B-form double-stranded DNA (dsDNA) molecules, comprising two complementary single strands of DNA (ssDNA) assembled by hybridization through Watson–Crick base pairing [12]. They exhibit a right-handed double-helix structure (2 nm diameter) with a periodicity of 10.5 bases and a distance of 3.4 Å between each base pair [1,12]. Leveraging the sequence specificity and unique structural features of dsDNA along with the structural predictability of DNA assembly has facilitated the rapid development of the structural DNA nanotechnology field [11,13,14,15], and particularly enabled the emergence of the DNA origami method [16]. This latter method rapidly became the strategy of choice for synthesizing discrete nanometer-scale particles, notably enabling the assembly of custom complex 1-, 2-, and 3D discrete DNA nanoarchitectures with highly defined shapes and sizes [16,17,18,19,20]. DNA origami nanoparticles are assembled by folding a long ssDNA scaffold strand with an excess of several short complementary oligonucleotides (‘staple strands’) in a one-pot thermal annealing reaction (Figure 1a). These nanoparticles are now widely used in many biomedical applications, such as nucleic acid delivery [20,21], vaccine platform development [22,23,24], drug delivery [25,26,27], and cancer therapy [28,29,30,31], among others [32,33,34]. 

Unlike other nucleic-acid-based nanoparticle assembly techniques that rely on the equimolar assembly of short oligonucleotides [35,36], the complexity and size of the assembled DNA origami nanostructures mainly depend on the scaffold strand length, sequence, and method of production [37] (Figure 1b). Furthermore, the amount of DNA origami nanoparticles that can be assembled will depend on the scaffold availability. DNA origami nanoparticles are commonly assembled using the M13mp18 bacteriophage’s genome—a commercially available 7249 nucleotide (nt)-long circular single strand of DNA—which can readily be used to assemble nanoparticles in a 10 to 100 nm size range [16,17,18,19,38]. However, the emergence of several new types of design software [19,39,40,41,42] enabling the automated design of complex nanostructures with any shape or size—and the increasing number of biomedical applications [43,44,45] have led to the increased complexity and size of designed DNA origami. The standard M13mp18 scaffold strand may limit the sizes of these newly designed nanoarchitectures. In addition, the sequence of the scaffold might affect the performance of the DNA origami nanostructures for a given application. For instance, recent works suggest the importance of sequence design to ensure immunocompatibility of the DNA nanoparticles [46]. Thus, controlling the sequence of the scaffold strands to avoid the presence of phage genes that might have an undesirable effect for in vivo applications, or controlling the presence or absence of immunogenic CpG domains is necessary. For all these reasons, the use of M13mp18 ssDNA as the sole source of scaffolding for DNA origami synthesis is now becoming a limiting factor. To design custom nanostructures and precisely control their sequences, establishing novel and efficient custom ssDNA scaffold synthesis methods is crucial to leverage DNA origami’s full potential. To be sustainable for biomedical applications, the production scale of ssDNA scaffolds also needs to be drastically increased to reduce the costs of production and enable a broader range of applications.

This review focuses on existing and emerging techniques for the synthesis of ssDNA scaffolds for DNA origami folding. In particular, it describes the various bacteriophage production methods, enzymatic synthesis strategies, and highlights promising new approaches to further develop the existing toolbox for scaffold synthesis. The methodologies, yields, functionality, and limitations of each method are presented herein.

## 2. Current Methods for ssDNA Scaffold Production

### 2.1. Bacteriophage-Based ssDNA Production

**Figure 2:**
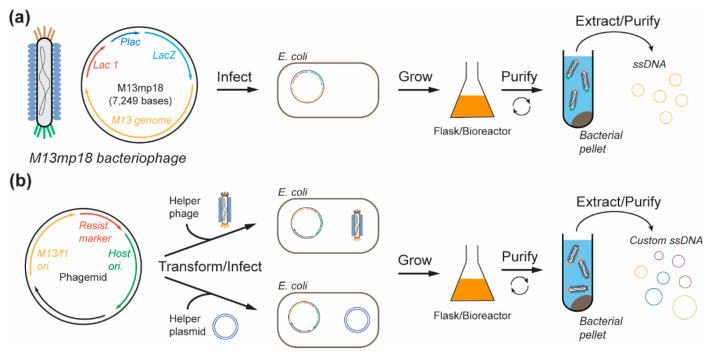
Bacteriophage-based methods for DNA origami scaffold production. (**a**) M13mp18 is used to infect *E. coli* bacteria where it can replicate and form progeny phages that are released into the culture medium, extracted, and purified to yield single strands of DNA (ssDNA) scaffolds. (**b**) Custom ssDNA scaffolds (lengths and sequences) are encoded into phagemid along with other essential genes for replication. The phagemid is used to transform the host *E. coli* in the presence of a helper phage or a helper plasmid. The transformed/infected host cells are grown, and the extruded progeny phages containing target ssDNA are extracted from the medium.

In the past few decades, ssDNA has been mainly used for specific biotechnological applications, such as cloning, sequencing, and phage display [47,48,49]. For these different applications, the most common cost-effective source of ssDNA is the circular genome of the filamentous bacteriophage M13. This bacteriophage—whose genome can be deftly and easily engineered—infects Escherichia coli (*E. coli*) and then replicates to produce progeny phages that extrude directly into the culture medium without causing bacterial lysis. The progeny phages are then extracted, and the ssDNA genome is purified and ready to be used as a source of ssDNA (Figure 2a). Moreover, given that the circular ssDNA (cssDNA) genome of M13mp18 is an engineered version of the bacteriophage M13, which offers a higher replication rate [50], it naturally became the first source of the ssDNA scaffold used for DNA origami assembly by Paul Rothemund in 2006 [16]. Currently, M13mp18 and a few of its length and sequence variants [50,51,52] remain the main sources of ssDNA scaffolds for DNA origami assembly. 

However, since M13mp18’s first application as a DNA origami scaffold, significant progress has been made in improving its production process and facilitating the development of biomedical applications. While M13mp18 scaffolds were originally produced in simple shaker flask-based cultures with yields ranging from 1 to 14 mg/L of culture [53,54,55] (Table 1), optimized methods have drastically increased this yield while significantly reducing the volume of the culture required. Kick et al. [56] were notably able to produce an ssDNA scaffold from the bacteriophage M13mp18 and two of its length variants (7560 and 8604 bases), with yields ranging from 370 to 410 mg/L of culture (Table 1) [52]. These yields were achieved using a high-density bacterial culture under controlled culture conditions (pH, substrate availability, and dissolved oxygen concentration). More recently, the same group further improved their high-cell-density fermentation method by finely tuning the time of infection, the cell-specific growth rate, and the multiplicity of infection (the ratio of phage used to infect a culture of a host bacteria at a given time) to produce M13mp18 ssDNA with a yield of 590 mg/L [57] (Table 1).

While the M13mp18 scaffold can be produced at a large-scale, its genome contains genes and regulatory sequences necessary for ssDNA replication, packaging, and extrusion into the culture medium, inherently limiting the final sequence and the minimum size of the ssDNA scaffolds produced. Thus, biomedical applications that require a specific sequence and/or length present a clear need for more flexibility in scaffold synthesis. The use of phagemids that can be produced in a similar manner to M13, but with more flexibility to create custom scaffolds, appears to be a good alternative to simple bacteriophage infection. Phagemids contain two origins of replication (called ‘ori’), one dsDNA ori and one ssDNA ori. The dsDNA ori corresponds to the plasmid origin of replication, for amplification of the phagemid, while the ssDNA ori that originates from an f1 bacteriophage or M13 phage is responsible for ssDNA phage replication (Figure 2b). The phagemid sequence itself does not encode for any M13 proteins. Thus, parallel infection with a helper phage is required to provide the viral components necessary to package the produced ssDNA into the progeny phage particles [58] (Figure 2b). While the helper phages conserve their ability to replicate in *E. coli*, they preferentially package the ssDNA encoded by the phagemid over their own ssDNA. Using this method, Zadegan et al. [59] infected *E. coli* with the phagemid pUC1983 and the helper phage M13KO7 to produce a 1983 nts ssDNA scaffold. This scaffold was later used to assemble a 18 × 18 × 20 nm hollow 3D DNA origami box, which is a common DNA origami structure used as a potential drug delivery system [60,61]. This method, with the same helper phage, was also applied by Li’s group [62], who designed four different phagemids encoding four distinct ssDNA scaffolds, each greater than 10,000 nts. After purification, these scaffolds were folded into multiple large 2D DNA origami nanostructures with edge sizes up to 300 nm. 

Although helper phages preferentially package the phagemid ssDNA, they can also replicate and package their own ssDNA genome that will be released into the culture medium, thus potentially contaminating the ssDNA production. To mitigate this problem, some studies have used helper plasmids instead of helper phages (Figure 2b). Brown et al. [63], for example, transformed an *E. coli* strain with a helper plasmid that encodes M13 coat proteins but does not contain the ssDNA origin of replication found in helper phages, thus enabling the packaging of the phagemid ssDNA into the progeny M13. The major advantage of this approach is that the helper plasmid is not replicated and packaged, which was the issue when using a helper phage, thereby avoiding the presence of contaminant DNA [64]. In the study by Brown et al., the authors developed a smaller vector system called mini-M13 (pSB4434), a variant of the phagemid pBluescript KS(-) commonly used for gene expression [65], and a helper plasmid (pSB4423) to produce a 2404 nts ssDNA scaffold with a yield of 0.2–0.4 mg/L (Table 1). The synthesized ssDNA was further used to assemble multiple 2D and 3D nanostructures [63]. Nafisi et al. [66] produced custom scaffolds of different lengths, ranging from 1512 to 10,080 bases, with a milligram-scale yield by using a custom phagemid and a helper plasmid (M13cp). The various scaffolds were subsequently used to fold brick-like structures and nanotubes [67].

In an effort to make a longer scaffold, LaBean’s group cloned the M13 phagemid pBluescriptKS(-) into bacteriophage λ to create an M13/λ hybrid phage called λ^M13^ [68]. Using the *E. coli* strain S3113, transformed with the helper plasmid (pSB4423) and infected with the λ^M13^ phage, the authors generated an ssDNA scaffold of 51,466 nts. This scaffold along with the conventional M13mp18 scaffold were used to fold discrete notched rectangular structures. To illustrate the impact of scaffold length on resulting structure size, the longer scaffold yielded a surface area that was seven times larger than the structure folded with the conventional M13mp18.

Although phagemid-based scaffold production is a cost-effective and scalable method to obtain custom-length ssDNA, phagemids also contain a double-stranded origin of replication usually derived from the plasmids pUC18 or ColE1 [59,63,66,68] that can affect the purity of the final ssDNA scaffold. Indeed, the dsDNA ori is required in earlier steps of propagation of the phagemid as a dsDNA plasmid. However, it can also be amplified in parallel with phage production, so the target ssDNA produced might also contain plasmid dsDNA contaminants [63]. Consequently, this method sometimes requires an extra step of purification. To solve this issue, Shepherd et al. [69] recently designed two miniphage genomes named phPB52 (1676 bases) and phPB84 (2520 bases), both containing an f1 single-stranded ori and the latter containing a custom synthetic insert of DNA to increase the size of the ssDNA fragment to be produced. The designed miniphage did not contain a double-stranded origin of replication, thereby avoiding contamination by dsDNA. Using the *E. coli* strain SS320 transformed with the helper plasmid M13cp [64], the authors produced pure cssDNA with no detectable dsDNA contamination. Using batch fermentation, they obtained a yield of 2 mg/L of pure cssDNA (Table 1), which is comparatively lower than that of the optimized ssDNA production using the M13mp18 phage [53,54,55] but with a higher percentage of purity. They further used the custom cssDNA scaffold to assemble monodisperse pentagonal bipyramid DNA nanoparticles with high folding efficiency. This structure was notably used to assemble antigen presenting nanoparticles with nanoscale precision [24].

With advances in biotechnology, scalable methods for the production of ssDNA scaffolds using phagemids are emerging and could potentially change the way DNA origami is produced for biomedical applications. For example, a study by Praetorius et al. [70] described a phagemid that simultaneously encodes for both the scaffold and staple ssDNA (Table 1), which were purified and then used for a one-pot assembly of DNA nanorods. The total yield of the folded DNA nanorods was reported to be 163 mg. In a following study from the same group, Engelhardt et al. [71] reported a sequence design method that used a split-ori phagemid to generate custom sequence-controlled scaffolds of different lengths, which were used to assemble the 42 helix-bundles lacking CpG motifs. This exclusion of CpG motifs may dampen the CpG-induced immune response by avoiding Toll-like receptor-9-mediated immunogenic reactions [72], thus facilitating the in vivo use of DNA origami for biomedical applications.

### 2.2. PCR-Based Methods for ssDNA Production

**Figure 3:**
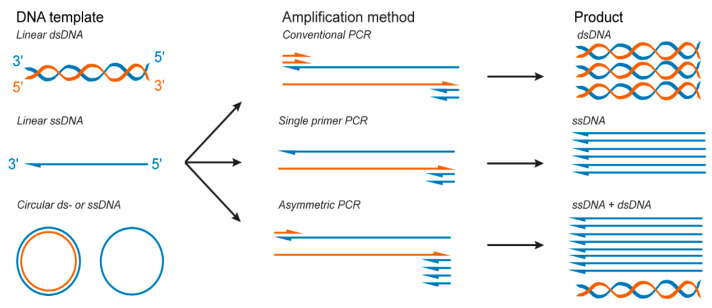
PCR-based methods to produce double-stranded DNA (dsDNA) and ssDNA.

Polymerase chain reaction (PCR) is a key method used in molecular biology that enables the amplification of target DNA sequences. Classic PCR allows for the exponential amplification of target dsDNA strands from various DNA templates using an equimolar concentration of forward and reverse primers [73] (Figure 3). 

While a few studies have reported DNA origami folding from dsDNA scaffolds [74,75], DNA origami folding is typically performed with ssDNA scaffolds. Thus, dsDNA products synthetized by classical PCR require additional steps of separation and purification to produce ssDNA that can serve as a scaffold strand for DNA origami folding. To this end, as previously established, for example, for ssDNA aptamers synthesis, various methods have been further developed to obtain pure ssDNA scaffolds from dsDNA PCR products, such as denaturation and separation with streptavidin magnetic beads [76,77,78], capture electrophoresis [79], and preferential DNase digestion of one of the strands [80,81,82,83]. Alternatively, studies have used single-primer PCR [84] and asymmetric PCR (aPCR) [85] (Figure 3), two variants of the PCR technique that allow for the direct production of ssDNA that can be isolated from dsDNA byproducts via agarose gel extraction and used without further purification. 

#### 2.2.1. Purification Methods to Produce ssDNA from Amplified dsDNA PCR Products

**Figure 4:**
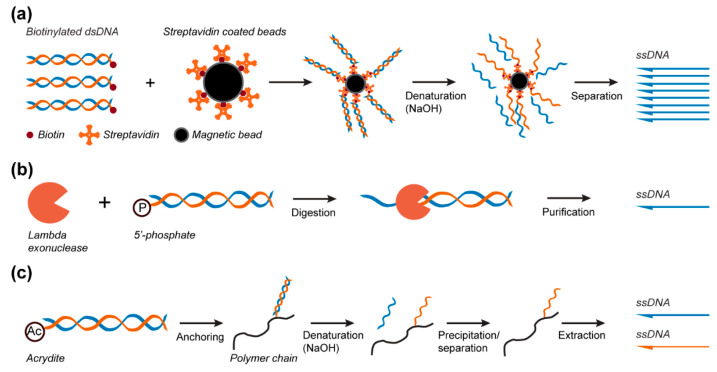
ssDNA scaffold production from PCR products. (**a**) A biotin-modified primer is used in PCR amplification for further biotin–streptavidin magnetic bead immobilization, dsDNA denaturation, and ssDNA separation. (**b**) Incorporation of a phosphate group in the PCR amplification allows for the subsequent generation of ssDNA scaffolds by the preferential DNase digestion of the PCR product. (**c**) Schematic of the selective nascent polymer catch-and-release (SNAPCAR) method. In this technique, an acrydite-modified dsDNA product generated by PCR is anchored to a linear polymer chain for purification of the ssDNA scaffolds. After separation, both ssDNA strands can be recovered.

*Biotin–streptavidin magnetic bead purification.* In this method, one of the primers is modified at its 5′ end with one biotin [76] or a dual-biotin group [78] to asymmetrically biotinylate the dsDNA product. Following PCR amplification, the biotinylated dsDNA product is captured by streptavidin-coated magnetic beads (Figure 4a) and precipitated by a magnetic force, and the supernatant is then exchanged to remove the excess primers, deoxynucleotide triphosphates (dNTPs), and enzymes. This step is followed by alkaline denaturation of the dsDNA product with sodium hydroxide [76,86] to separate the two DNA strands. The magnetic beads are again precipitated by magnetic force to spatially segregate the two strands, and the free ssDNA is simply recovered after pH neutralization [76] (Figure 4a). Pound et al. used this extraction method to synthesize ssDNA scaffolds of 756 and 4808 nts from dsDNA PCR products. The produced scaffolds were folded into various thin and branched letter-shaped DNA origami with dimensions up to 250 nm [76]. However, the harsh conditions used during the process of denaturation of the dsDNA can affect the streptavidin–biotin interactions, which might result in dsDNA contamination in the final product [87]. Additionally, because an excess of biotinylated primers are used in the PCR reaction, magnetic beads are quickly saturated, thus requiring one to use high quantities of beads to capture all ssDNA strands for most of these protocols, making this strategy an expensive method.

*Preferential DNase digestion.* Lambda exonuclease is the most common enzyme used to produce ssDNA from dsDNA PCR products. This enzyme can bind to dsDNA and selectively digest the DNA strand bearing a 5′ terminal phosphate group, which is incorporated during PCR amplification via the use of modified primers [88] (Figure 4b). Using this method, Zang et al. [83] were able to generate a 26,182 nts ssDNA scaffold from a dsDNA fragment amplified by PCR with the lambda phage genome used as a template. This ssDNA scaffold strand was then used to fold a very large 2D rectangle-shaped DNA origami with dimensions of 239.6 nm x 108.6 nm [83]. In another study using this method, Han et al. [89] prepared self-complementary ssDNA scaffolds with sizes ranging from 966 to 10,682 nts for staple-free DNA origami assembly. This purification method requires optimization of the enzyme quantities and digestion time to obtain only ssDNA, as well as an extra step of purification to remove the enzyme [80]. Although effective to eliminate the enzyme, this additional purification step is also associated with a loss of ssDNA [81,82] and is sometimes ineffective for separating residual dsDNA from ssDNA [90]. In addition, the cost of the enzyme used for this method might become a limiting factor for its adoption as a large-scale production method [90].

*Selective Nascent Polymer Catch-and-Release* (SNAPCAR). A promising new approach for the direct production and extraction of ssDNA is selective nascent polymer catch-and-release [91]. This method uses a linear poly(acrylamide-co-acrylate) chain to capture acrydite-modified dsDNA strands synthetized by classical PCR. As seen in biotin-streptavidin magnetic bead separation, NaOH is added to denature the dsDNA product and release the non-anchored target strand into the solution. The polymer and bound strand can then be precipitated to enable extraction of the target ssDNA strand (Figure 4c) [91]. SNAPCAR was further developed into methanol-responsive polymer PCR (MeRPy-PCR) [92] to enable the subsequent extraction of the anchored ssDNA strand. This was achieved by the inclusion of an uracil base in the acrydite-modified primer. The non-anchored strand is extracted as in SNAPCAR; however, the anchored strand is then released by cleaving the uracil base through subsequent incubations with uracil-DNA glycosylase (UDG) and dimethylethylenediamine (DMEDA). The cleaved ssDNA strand is then extracted by precipitation of the polymer anchor (Figure 4c). These methods were able to produce ssDNA up to 7308 nucleotides in length. The produced scaffold was used to fold DNA origami barrels, plates, and rods [91,92].

#### 2.2.2. Asymmetric Polymerase Chain Reaction for the Direct Production of ssDNA

aPCR is one of the most extensively used methods for the direct production of short ssDNA aptamers [90,93,94,95,96]. Due to its highly specific reaction conditions and several limitations, this method was originally limited to the production of ssDNA with ten to a few hundred bases [94,97]. However, recent studies have optimized the reaction conditions, enabling synthesis of long ssDNA scaffolds to fold DNA origami [24,98,99]. In contrast to classical PCR, aPCR allows the direct synthesis of ssDNA from any dsDNA or ssDNA template and does not require any specific method to separate the dsDNA products [19,73,84,85,93,98] (Figure 3). In a standard aPCR reaction, an asymmetric concentration of primers can be utilized [85] to amplify a specific template (Figure 3). Specifically, a reduced amount of the primer that amplifies the complementary strand is used to generate the secondary template with the correct length, from which the ssDNA of interest is amplified with the excess primer. The primer concentration and primer ratio are two of the major factors that influence the final ssDNA production yield [85,93]. This strategy can generate specific templates from any DNA sequences in a single reaction, unlike the single-primer PCR strategy. Veneziano et al. notably optimized the aPCR reaction parameters (the ratio of primers, number of cycles, and polymerase type) to improve the yield and purity of ssDNA produced with sequence lengths up to 15,000 nts via the aPCR method [85]. These custom scaffolds have been used to fold several DNA origami nanoparticles with a wide range of geometries and are now used to produce DNA origami-based antigen-presenting nanoparticles [19,24,85]. Moreover, as for classic PCR, aPCR can be used to introduce modified nucleotides during ssDNA synthesis, which could facilitate the production of modified scaffolds for DNA origami and enable direct functionalization of the DNA origami. For instance, aPCR-produced ssDNA scaffolds were successfully synthetized with phosphorothioate bonds that can improve the stability of the DNA origami against nuclease degradation and fluorophore modification to improve tracking of the nanoparticles [85]. 

### 2.3. Alternative Enzymatic Methods for ssDNA Scaffold Production

In addition to PCR-based methods that usually involve purification steps and limited production yields, few alternative enzymatic methods have been developed for the production of full-length ssDNA scaffolds, with notable examples including rolling circle amplification (RCA) and sequential growth, among others [100,101,102,103,104].

#### 2.3.1. Rolling Circle Amplification

**Figure 5:**
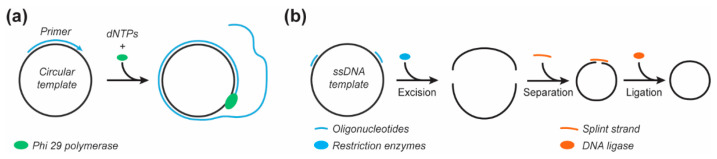
Alternative enzymatic methods for ssDNA scaffold production. (**a**) Rolling circle amplification. (**b**) Excision and circularization of a ‘mini’ scaffold from the M13mp18 genome.

RCA enables the isothermal amplification of long ssDNA concatemers up to several thousand nucleotides long, containing from ten to a few hundred tandem repeats complementary to the template sequence [105]. This method yields up to milligram quantities of pure ssDNA from a simple overnight reaction [106]. The RCA method requires a circularized ssDNA template, a single primer, and a polymerase with strand displacement capabilities, commonly the Phi29 (Φ29) polymerase purified from the *Bacillus subtilis* bacteriophage Φ29. The polymerase begins extending the primer around the circular template strand to form a complementary strand. Once the starting point is reached, the polymerase begins to displace the 5′-end of the newly synthesized strand, permitting the polymerase to continually synthesize the concatemeric ssDNA for up to 8 h in a single reaction [107] (Figure 5a). The strength of this method is its simplicity and the large amount of ssDNA produced. However, the use of concatemeric ssDNA scaffolds is limited to nanostructures that include repeated motifs, limiting the monodispersity of the assembled architectures and not allowing for discrete nanoparticle assembly. This method has been used to fold structures including DNA nano-wires/plates [108] or ladder assemblies [109]. These concatemeric structures also appear to be useful as passive carriers for intercalating drugs and CpG motifs [110], though they lack the resolution for logic-gated or dynamic approaches for drug-delivery and immunotherapies. Another study by Yan et al. highlighted some specific advantages of RCA-produced scaffolds to increase the sensitivity in biosensing applications. They designed nanostructures containing repeated motifs to conjugate multiple horseradish peroxidase (HRP) enzymes in order to amplify the signal of a prostate-specific antigen detection assay [111]. The simplicity of RCA and the resulting high yield warrant efforts toward sequestering the repeated sequences into discrete ssDNA strands for folding more complex structures.

Recent advances demonstrated the ability to cleave RCA-produced concatemeric ssDNA into shorter ssDNA strands by programming restriction enzyme sequences (*Sma*I/*Hind*III) into the scaffold on either side of the target ssDNA and performing post-amplification digestion [106]. One drawback to this approach is the need to sequentially digest each restriction site, including an intermediary purification step and complementary *Sma*I/*Hind*III oligos to recognize the restriction enzyme site. A similar approach used hairpin-forming BseGI recognition sequences between the target ssDNA. This approach was used to produce short ssDNA strands from 14 to 378 nts [112]. To alleviate some of the sequence specificity and enzymes required for the digestion of long ssDNA into programmed fragments, Zn-dependent DNAzymes have also been implemented [70,113]. These methods can be applied to isolate discrete ssDNA scaffolds and reduce the design limitations of classic RCA-based scaffolds, to leverage the high production yield of the RCA method.

#### 2.3.2. Sequential Growth of ssDNA

More recently, sequential growth of an ssDNA scaffold was achieved through the temporal assembly of multiple synthetic dsDNA blocks (42 nts) [102]. Each of the dsDNA blocks were synthesized with single-stranded sticky overhangs (10 nts) programmed at their 5′ or 3′ ends to anneal with a complementary sticky overhang of the subsequent building block. The annealed dsDNA blocks were ligated with T4 DNA ligase to produce a long linear dsDNA strand (458–1058 bps). The respective termination building blocks exhibited blunt-ends, and the resulting custom dsDNA product was amplified by classical PCR. Streptavidin–biotin magnetic bead separation was used to isolate the ssDNA product similar to the previously described method in this review. Scaffolds produced by sequential growth were used to fold DNA nanotubes, which served as the template for 15 nm streptavidin-coated quantum dots [102], as well as to create ‘railroad tracks’ to join DNA origami plates for the organization of higher-order structure assembly [114]. This scaffold synthesis method offers the ability to produce a scaffold with an arbitrary sequence, whereas other methods are limited to existing biological templates and/or the incorporation of enzyme-specific recognition sites. However, this method requires multiple steps and a subsequent PCR-based approach to produce a sufficient quantity of ssDNA.

#### 2.3.3. Restriction Enzymes to Prepare a Smaller Scaffold

The adaptation of naturally sourced DNA may help expand the functionality of the produced scaffolds while maintaining considerable yields. A prime example was the production of a small circular ‘M1.3’ scaffold [104]. The M1.3 scaffold (704 nts) was obtained by digestion with restriction enzymes of the commonplace M13mp18 scaffold (Figure 5b). The linear M1.3 scaffold was then circularized by splint hybridization and subsequent ligation of the M1.3 fragment by the T4 DNA ligase before being successfully folded into various DNA origami structures. While this method is efficient for short ssDNA fragment production, the need for unique restriction endonuclease sites, as well as the production yield, might limit its use to specific applications.

## 3. Emerging Enzymatic Methods for ssDNA Synthesis

Alternative ssDNA synthesis methods continue to be developed and show promise toward potentially synthesizing full-length DNA origami scaffolds [115]. These emerging methods include nicking strand displacement amplification (nSDA), primer exchange reaction (PER), and terminal deoxynucleotidyl transferase-based (TdT) synthesis. While these methods show great promise, they will need to be further optimized before being used for DNA origami scaffold synthesis. In this part of the review, we describe the potential advantages and limitations of these techniques.

### 3.1. Nicking Strand Displacement

**Figure 6:**
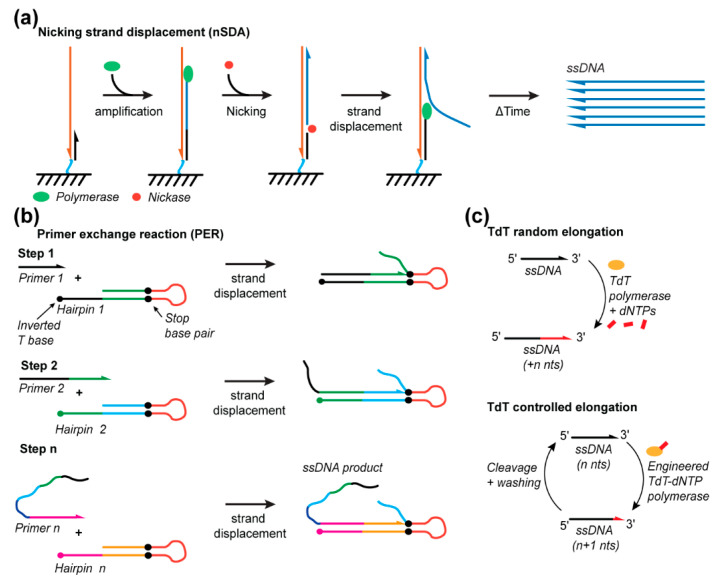
Emerging enzymatic methods for ssDNA synthesis. (**a**) Nicking strand displacement (nSDA) technique. (**b**) Primer exchange reaction for programmable synthesis of ssDNA. (**c**) Terminal deoxynucleotidyl transferase methods to elongate (random) ssDNA or for de novo synthesis (controlled elongation).

nSDA offers a simple means to amplify and extract ssDNA strands. A recognition sequence is programmed into a single primer for nicking endonuclease to cleave the DNA backbone at a specific point in the sequence/endonuclease, thus producing a nick and permitting strand displacement amplification (Figure 6a). The nSDA method utilizes a strand-displacing polymerase, such as the Bst-Large Fragment, and a nicking endonuclease, such as Nt.BstNBI, to nick the recognition site. The region beyond the nick is replicated by displacing the existing strands from the template strand, simultaneously producing, and releasing ssDNA into solution (Figure 6a). This method was employed on a microfluidic chip with anchored template oligos containing a universal primer sequence that encodes the nicking endonuclease recognition site, beyond which the template of the sequence to be amplified is encoded. The anchored strands were linearly amplified from 2, 3, and 4 ng of the template DNA to release approximately 7, 11, and 14 ng, respectively, synthetized 48 nt ssDNA strands into solution [116]. Additionally, the chip was shown to be reusable for at least 10 amplification protocols. This yield could be improved by using a chip with a micropillar array to improve the efficiency of mass transport near the anchored strands and/or by subsequent amplification of the produced strands via PCR [68]. A similar strategy used for gene assembly incorporates on-chip polymerase chain assembly (PCA) after nSDA to synthesize custom-sequence dsDNA strands with lengths of 500 to 1000 nts for efficient assembly yield but with the theoretical capacity to produce ~30,000 nts products from 10,830 different 85 nts oligonucleotides produced by nSDA [117]. By using different enzymes, Sequenase 2.0 and the nicking endonuclease Nt.BspQI, and the E. Coli single-stranded binding protein (SSB) to stabilize the longer ssDNA products, 500, 1000, and 5000 nt ssDNA templates were linearly amplified for 40 min to produce approximately 68, 55, and 180 ng of product, respectively, from 2 nM of a DNA template in a 5 μL reaction [118]. Thus, nSDA offers great potential for rapid DNA origami scaffold production.

### 3.2. Primer Exchange Reaction

PER involves the programmed sequential extension of seed ssDNA through multiple DNA hairpin primers/templates [119,120]. Each hairpin consists of an open primer region (~7–9 nts) and a self-complementary amplification region (~9 to 14 nts). Amplification is confined to only the template region by incorporating inverted bases and synthetic base pairs. The final sequence of a template region encodes the priming region for the next hairpin in the programmed sequence (Figure 6b). This methodology controls the sequential extension of ssDNA that can be used for the synthesis of ssDNA staples used in DNA origami or implemented as a micro-RNA-detecting logic gate. Forty different staple strands (~32 nts) were synthesized in a single reaction, thereby demonstrating the specificity of this approach, and an individual five-step cascade produced a 60 nts oligo. A repeating telomeric sequence was also produced in the order of a few hundred nucleotides [120], supporting the potential for this approach to be developed for scaffold production.

### 3.3. Terminal Deoxynucleotidyl Transferase

TdT is another promising new method enabling the de novo enzymatic synthesis of ssDNA strands, but is currently limited to the synthesis of very short ssDNA fragments [121,122] (Figure 6c). TdT is an enzyme that naturally adds nucleotides to the 3’ end of ssDNA, though it does so indiscriminately and, thus, without control over the number of bases added [123,124]. Recent developments anchored a single nucleotide to the enzyme for the controlled addition of a single base [122]. This base is anchored to the enzyme with a cleavable linker such that UV-exposure can release the enzyme from the extending ssDNA strand following single-base addition. TdT has only been shown to produce 10 nts fragments thus far [122]. However, automation, optimization of the metal-ion cofactors [125], cleavable linkers, and engineering of the enzyme offer multiple degrees of freedom to significantly improve this yield and, thus, the length of ssDNA that can be synthesized by TdT.

## 4. Long ssDNA in Biomedical Applications beyond DNA Origami Folding

Beyond the synthesis of ssDNA as scaffolds to assemble DNA origami nanocarriers for drug delivery and cancer immunotherapy, a few other biomedical applications, such as aptamer production, hydrogel synthesis, imaging, and synthetic biology [126,127,128,129], would benefit from custom ssDNA synthesis technologies. One of the main potential applications for long ssDNA is genome editing, particularly the homology-directed repair (HDR) strategy that combines the clustered regularly interspaced short palindromic repeats (CRISPR)/Cas9 system with nucleic acid donor templates to perform genome editing [130,131,132,133]. Originally, due to the complexity of long-ssDNA-strand synthesis and the limitations of chemical synthesis [134], DNA was only utilized for short insertions (100–200 bases), while dsDNA donors were utilized for insertions of lengths greater than 100 bases [135,136]. However, recent studies have shown that ssDNA donor templates could have higher efficiency for HDR compared to dsDNA donor templates [137,138]. ssDNA donor templates are now being explored by multiple groups for genome editing. For example, Quadros et al. developed a technique called Easi-CRISPR for floxed, conditional, and insertion alleles with varying efficiencies of editing between the gene-insertion cassette; the efficiency of the editing varied from 25% to 100% [139]. Codner et al. further validated Easi-CRISPR’s effectiveness in generating conditional alleles and point mutations [129]. Thus, improved long-ssDNA production methods will be beneficial upon multiple fronts beyond DNA origami.

## 5. Conclusions

The potential of DNA origami nanoparticles has now been demonstrated through many successful biomedical applications, including drug delivery, vaccine platform development, and cancer therapy [19,20,21,22,25,26]. While the DNA origami field continues to grow rapidly, our limited capacity to produce custom scaffolds at a large scale is becoming a major roadblock, which is ultimately reducing the breadth and sustainability of DNA origami for biomedical applications. Thus, the need for scalable methods to produce pure ssDNA scaffolds with custom lengths and sequences is becoming crucial. In this review, we have presented the various strategies that have been developed, or are under development, to synthesize long ssDNA scaffolds for DNA origami folding. However, despite the rapid progress made in improving these strategies, they still suffer from limitations that must be solved to facilitate large-scale custom DNA origami production. For example, while bacteriophage-based production allows for the production of a large amount of ssDNA scaffolds in the milligram range, the flexibility in the sequence and length of the scaffold synthetized is limited compared to PCR-based methods. To readily produce sequence-specific scaffolds, PCR-based methods such as aPCR or single-primer PCR are favorable but yield a lower amount of the ssDNA scaffold. Some enzymatic methods yield a sizeable amount of ssDNA but require extra purification steps. Solving these challenges by developing new methods for de novo synthesis of long ssDNA scaffolds with custom length and sequence will certainly aid the development of early-stage biomedical applications and will facilitate the emergence of more applications that require highly specific DNA origami nanoparticles with tailored scaffolds.

## Figures and Tables

**Table 1:** Overview of bacteriophage-based ssDNA production.

Production Method	Phage/Phagemid Helper Phage/Helper Plasmid	Scaffold Size (nts)	Yield (mg/L)	Refs
Shaker flask	Phage (M13mp18)	7249	6.7–10 *	[54]
Bioreactor (High-cell-density)	Phage (M13mp18)	7249	410 *	[56]
		7560	370 *	
		8074	370 *	
Bioreactor (High-cell-density)	Phage (M13mp18)	7249	590 *	[57]
Shaker flask	Phagemid + Helper phage	10,563 10,782 21,261 31,274	1	[62]
Shaker flask	Phagemid + Helper plasmid	2404	0.2–0.4 *	[63]
Shaker flask	Phagemid+ Helper plasmid	1512 2268 3024 5544 8064 10,080	-	[66]
Shaker flask	Phagemid + Helper plasmid	1676	0.5	[69]
Stirred-tank bioreactor	Phagemid + Helper plasmid	2520	2	[69]
Shaker flask	Phagemid + Helper plasmid	2800 3200	4 *	[70]
Stirred-tank bioreactor	Phagemid + Helper phage	2800 3200	141 *	[70]
Shaker flask	Phagemid + Helper plasmid	1317 2873 4536 6048 7560 9072	0.38 3.6 ~5.7 ** ~4.2 ** ~1.4 ** 2.6	[71]

* Yields of ssDNA were converted to mg of ssDNA produced per L of culture (mg/L) from the original yield values reported in the corresponding references. ** Values are read from the figures in the references.
